# Supplementary material for: Measuring global human accessibility to essential daily necessities and services
Source: Nat Commun. 2025 Nov 28;16:10709. doi: 10.1038/s41467-025-65732-w (PMC12663381; doi:10.1038/s41467-025-65732-w)
Supplement: Supplementary file 1 — Supplementary Information [file 41467_2025_65732_MOESM1_ESM.pdf]

Supplementary Information for

Measuring global human accessibility to essential daily necessities and services

Shengbiao Wu[1], Bin Chen[1,2,3,*], Jiafu An[4], Andew Nelson[5], Fan Dai[3], Chen Lin[6], Peng Gong[2,3,7,*]

[1]Future Urbanity & Sustainable Environment (FUSE) Lab, Division of Landscape Architecture, Faculty of Architecture, The University of Hong Kong, Hong Kong SAR, China

[2]Urban Systems Institute, The University of Hong Kong, Hong Kong SAR, China

[3]Institute for Climate and Carbon Neutrality, The University of Hong Kong, Hong Kong SAR, China

[4]Department of Real Estate and Construction, Faculty of Architecture, The University of Hong Kong, Hong Kong SAR, China

[5]Department of Natural Resources, Faculty of Geo-Information Science and Earth Observation (ITC) of the University of Twente, The Netherlands

[6]Faculty of Business and Economics, The University of Hong Kong, Hong Kong SAR, China

[7]Department of Geography and Department of Earth and Planetary Sciences, The University of Hong Kong, Hong Kong SAR, China

*Corresponding author: Bin Chen (binley.chen@hku.hk) and Peng Gong (penggong@hku.hk)

List of supplementary items

Supplementary Figs. 1-28…………………Pages 3-31

Supplementary Tables. 1-3………………..Pages 32-34

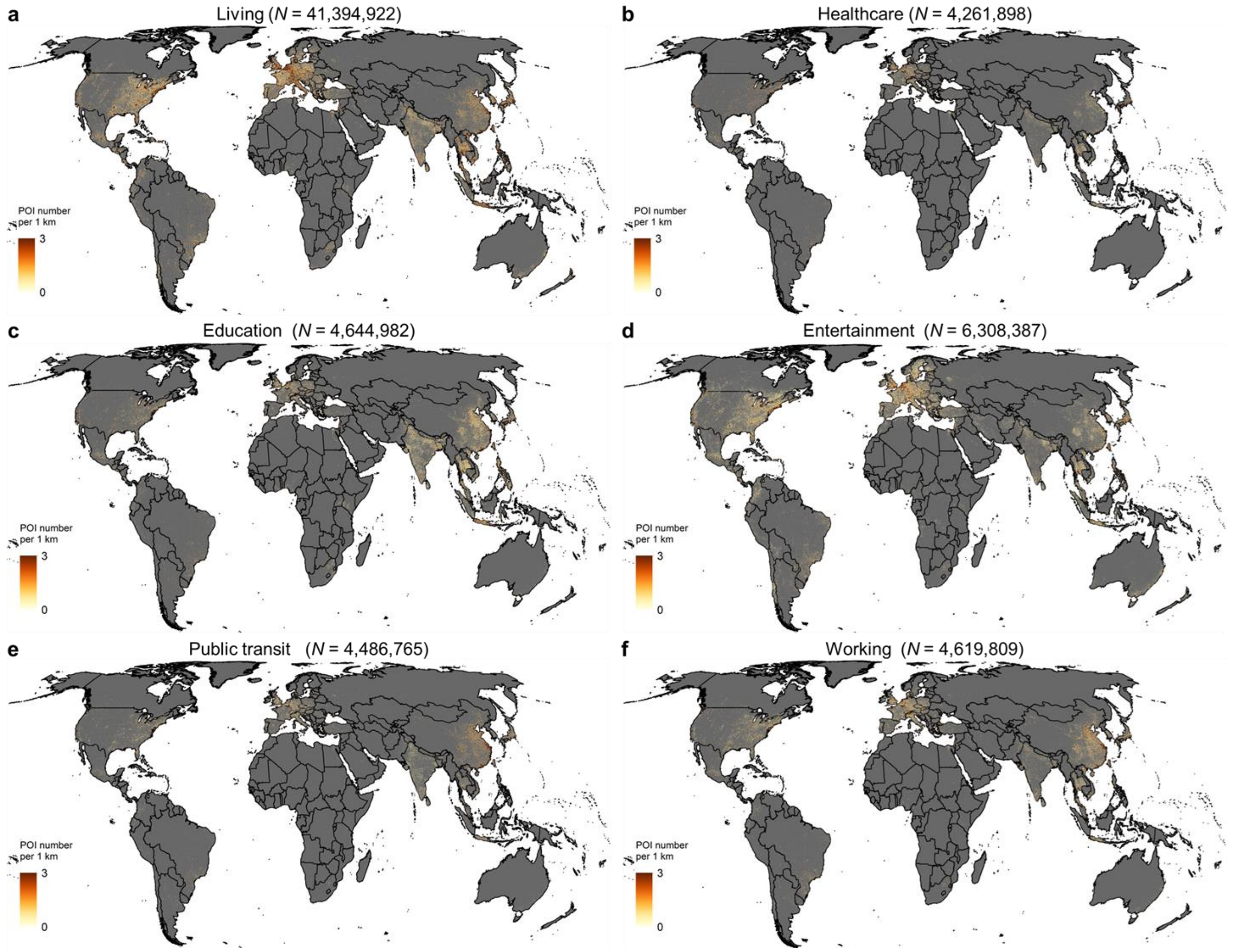

Supplementary Fig. 1. Density maps of individual point of interest (POI) number measuring six critical infrastructures in resident living services and amenities. **a-f.** POI density maps for living (**a**), healthcare (**b**), education (**c**), entertainment (**d**), public transit (**e**), and working amenities (**f**). The gray state boundaries vector is from the Global Administrative Unit Layers (GAUL) dataset provided by the Food and Agriculture Organization (FAO) of the United Stations (https://data.apps.fao.org).

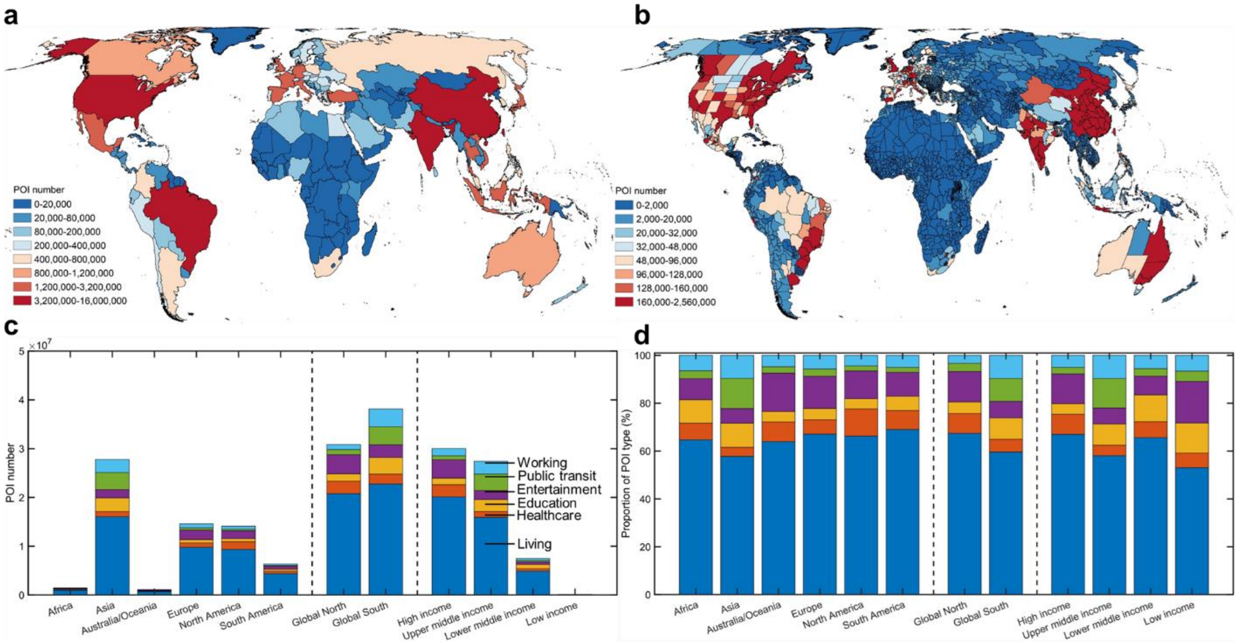

Supplementary Fig. 2. Distribution of point of interest (POI) number measuring six critical infrastructures across different spatial and socioeconomic settings. **a, b.** Spatial distributions of total POI number across country (**a**) and state (**b**) levels. **c, d,** Composition of different POI types in absolute (**c**) and relative (**d**) magnitude across different continental (Africa, Asia, Australia/Oceania, Europe, North America, and South America), urbanization (Global North and Global South), and income (high, upper middle, lower middle, and low incomes) contexts. Income group information is from World Bank database according to 2022 gross national income (GNI) per capita: low income, 1,135 USD or less; lower middle income, 1,136 - 4,465 USD; upper middle income, 4,466 - 13,845 USD; and high income, 13,846 USD or more. The boundaries vectors for country, state, continent, and Global North and Global South are from the Global Administrative Unit Layers (GAUL) dataset provided by the Food and Agriculture Organization (FAO) of the United Stations (https://data.apps.fao.org).

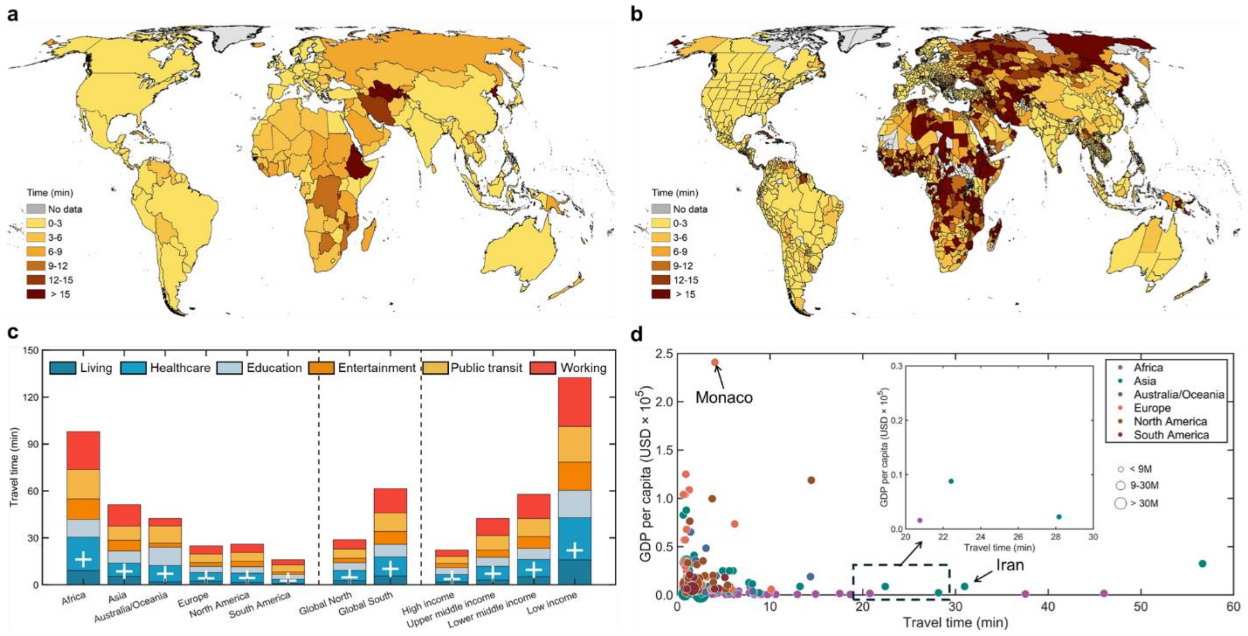

Supplementary Fig. 3. Global patterns of mean travel time for urban residents to their living services and amenities with access to motorized transport. a, b. Maps of mean travel time for urban residents' living services and amenities across country (**a**) and state (**b**) levels, where urban area is defined by global urban boundary (GUB) vector for 2020[33]. No data in **a-b** refers to countries or states without covering any GUB polygons. **c.** Travel time for six types of living services and amenities with motorized transport across different continental (Africa, Asia, Australia/Oceania, Europe, North America, and South America), urbanization (Global North and Global South), and income (high, upper middle, lower middle, and low incomes) contexts. The mean travel time for each continent, economic development, and income level are shown in each column as "+" symbol in white colour. Income group information is from World Bank database according to 2022 gross national income (GNI) per capita: low income, 1,135 USD or less; lower middle income, 1,136 - 4,465 USD; upper middle income, 4,466 - 13,845 USD; and high income, 13,846 USD or more. **d.** Relationship between the GDP per capita and mean travel time for urban residents, where the GDP data is available from the World Bank database. Two countries with contrasting GDP and travel times, Monaco and Iran, are highlighted and labelled by name.

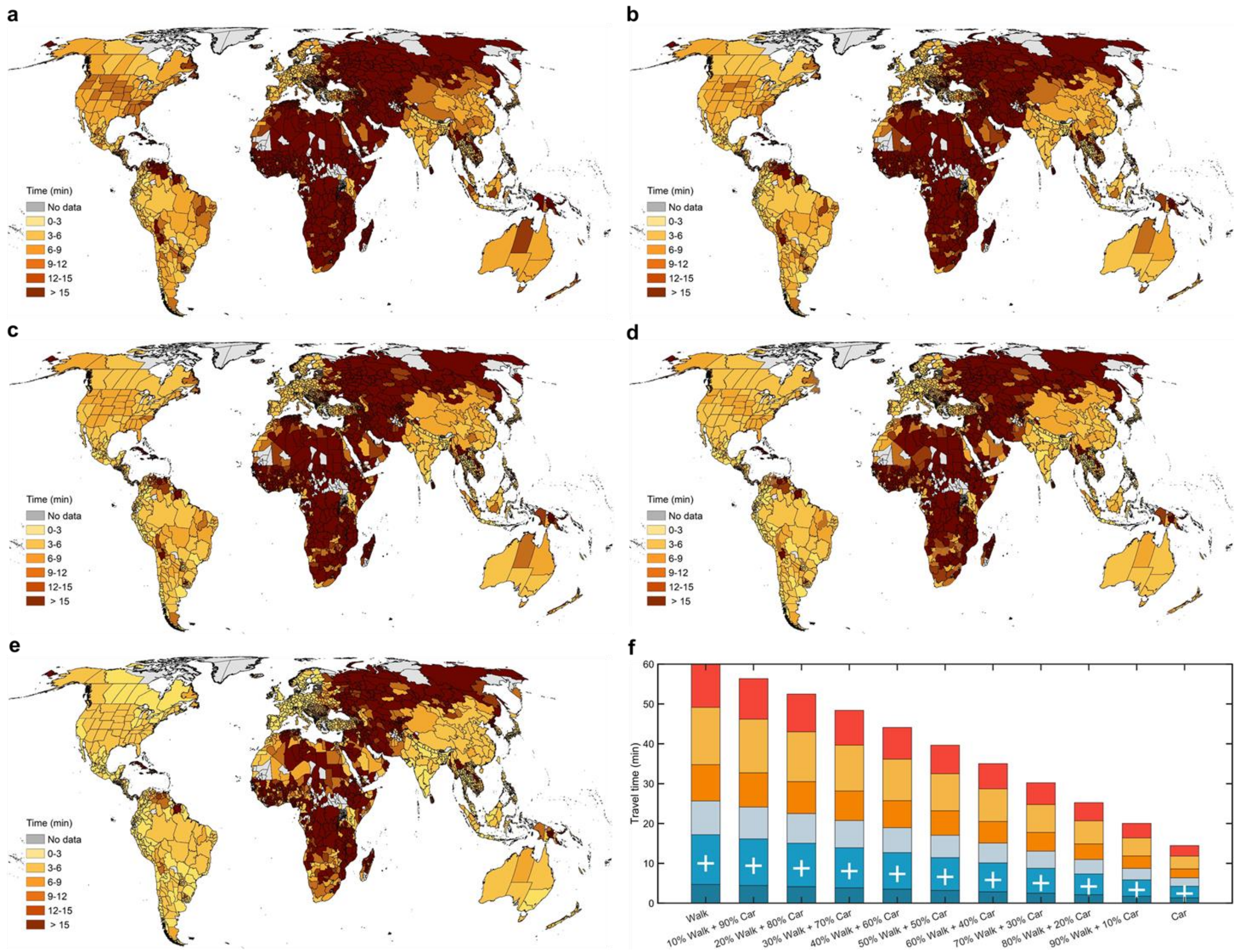

Supplementary Fig. 4. Global patterns of mean travel time for urban residents to their living services and amenities with access to a mixture transport. a-e. Maps of country-level mean travel time for urban residents' living services and amenities with 20% **(a)**, 40% **(b)**, 50% **(c)**, 60% **(d)**, and 80% **(e)** of walking-based transportation. **f.** Statistics of travel time for six types of living services and amenities across different mixture transportation modes. The mean travel time for different transportation schemes is shown in each column as "+" symbol in white colour.

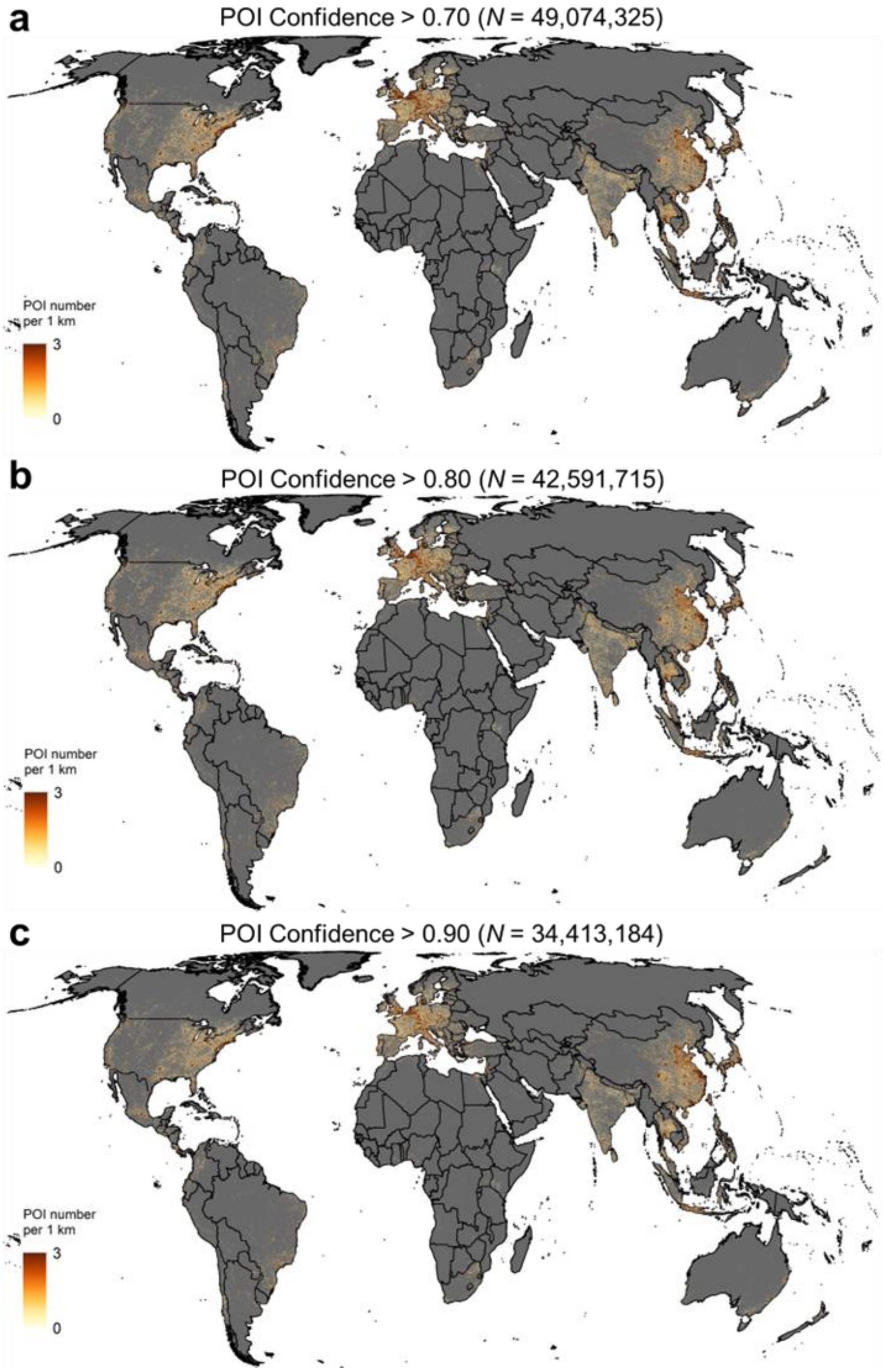

Supplementary Fig. 5. Density maps of total point of interest (POI) number of six critical infrastructures under different confidence levels. **a.** POI confidence level of 0.7. **b.** POI confidence level of 0.8. **c.** POI confidence level of 0.9. The gray state boundaries vector is from the Global Administrative Unit Layers (GAUL) dataset provided by the Food and Agriculture Organization (FAO) of the United Stations (https://data.apps.fao.org).

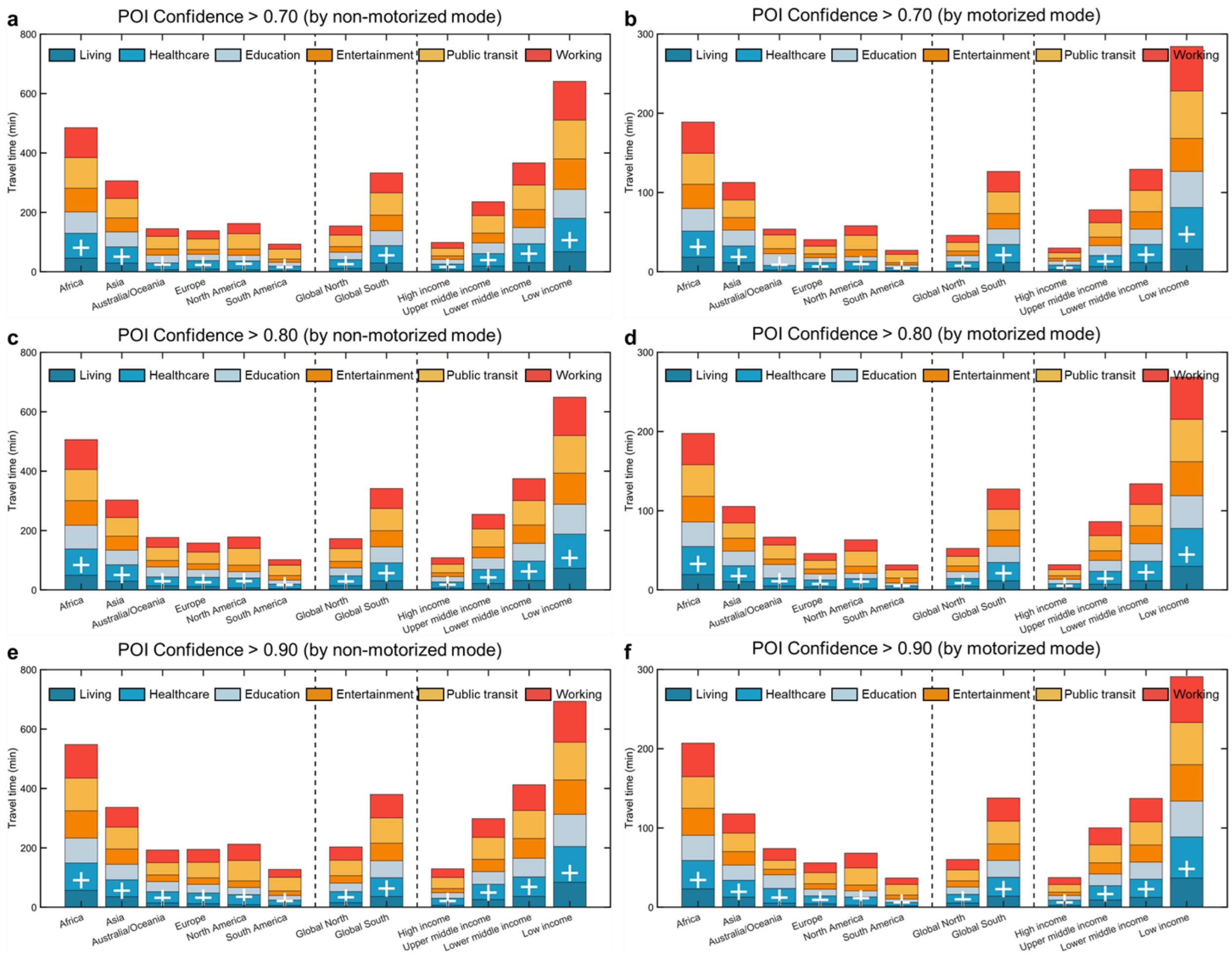

Supplementary Fig. 6. Sensitivity analysis of travel time for six types of living services and amenities by non-motorized (a, c, e) and motorized (b, d, f) modes across geography, socio-economic status, and income levels. **a, b.** Point of interest (POI) confidence of 0.7. **c, d.** POI confidence of 0.8. **e, f.** POI confidence of 0.9.

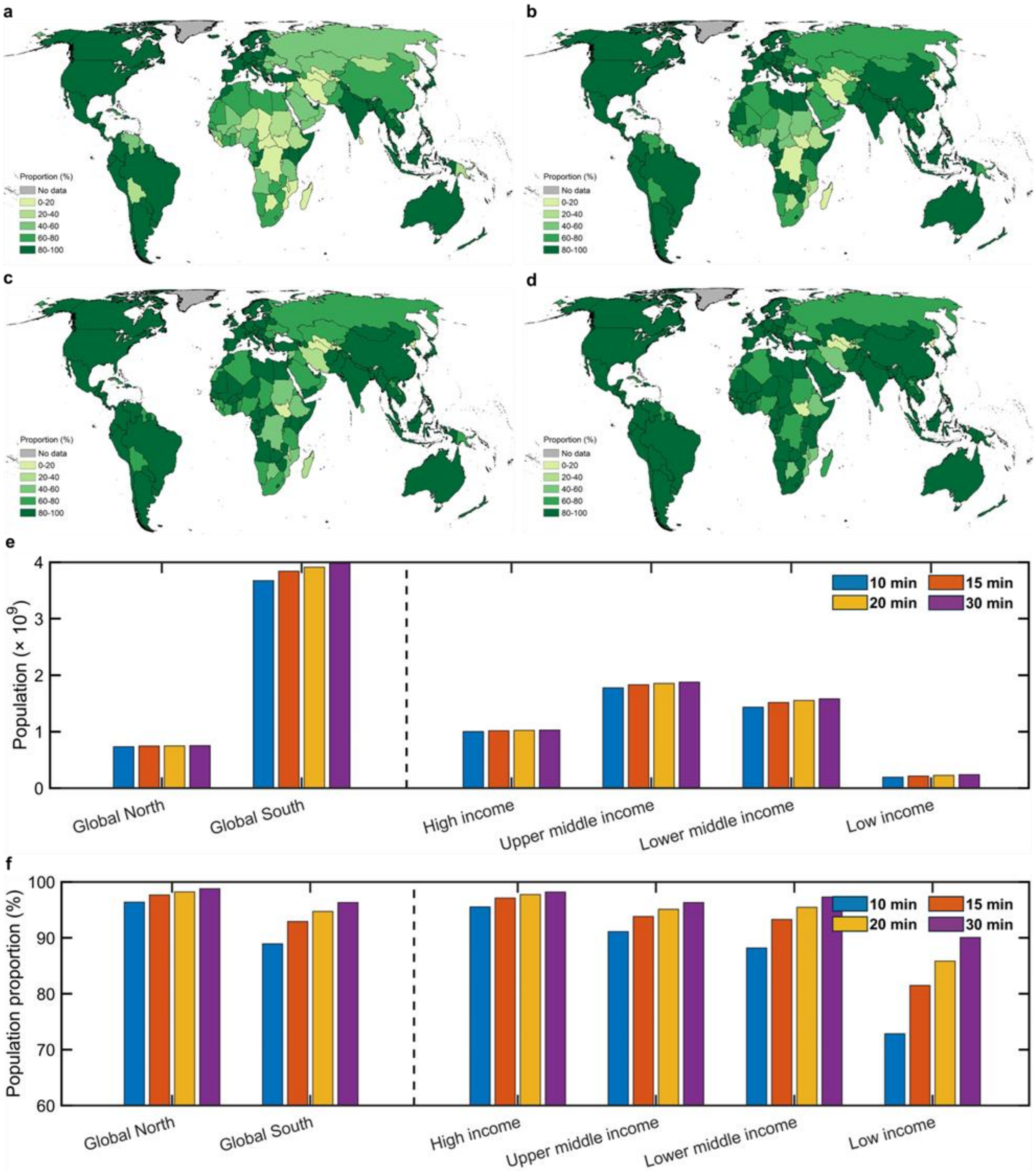

Supplementary Fig. 7. Urban population's accessibility to living services and amenities under the "*x*-minute city" concept with access to motorized transport. a-d, Maps of urban population proportion can reach their living services and amenities by a 10-minute (**a**), 15-minute (**b**), 20-minute (**c**), and 30-minute (**d**) drive. **e, f,** Absolute (**e**) and relative proportion (**f**) of urban population satisfy the "x-minute city" concept with motorized transport across different urbanization (Global North and Global South) and income (high, upper middle, lower middle, and low incomes) contexts.

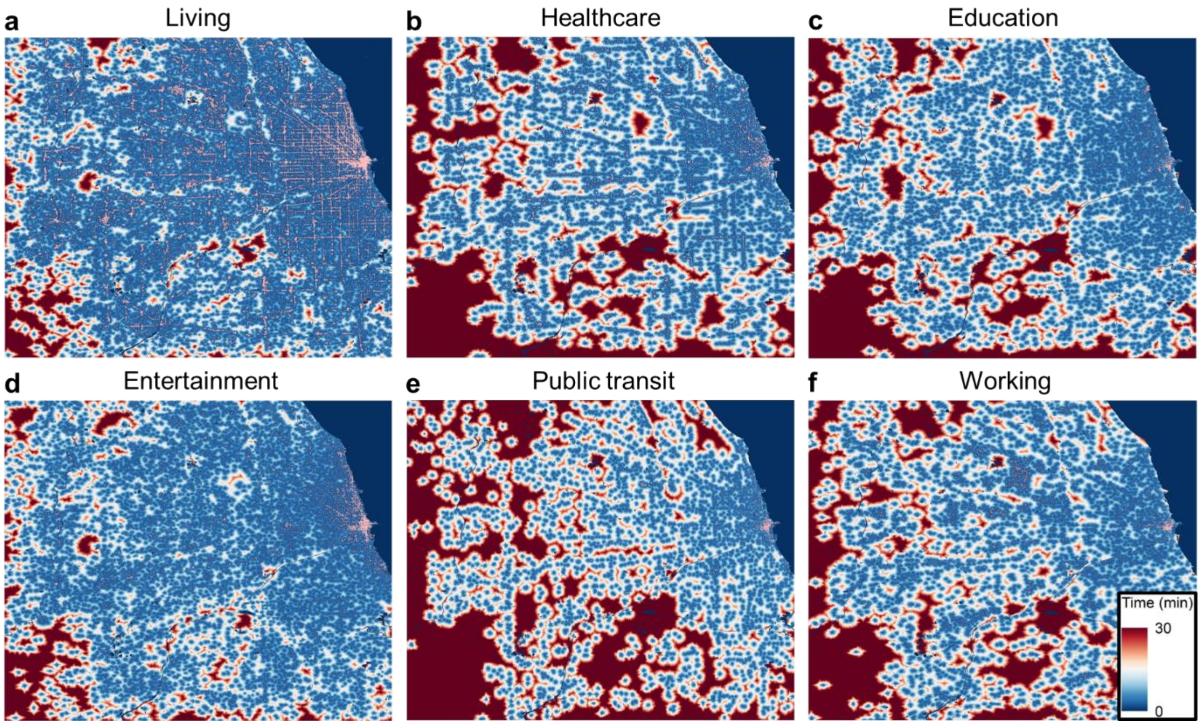

Supplementary Fig. 8. Local example of travel time to living services and amenities with non-motorized transport in Chicago, United States. a-f. Travel time maps for living (**a**), healthcare (**b**), education (**c**), entertainment (**d**), public transit (**e**), and working amenities (**f**). Point of interest (POI) datasets are overlaid on the travel time map with purple colours.

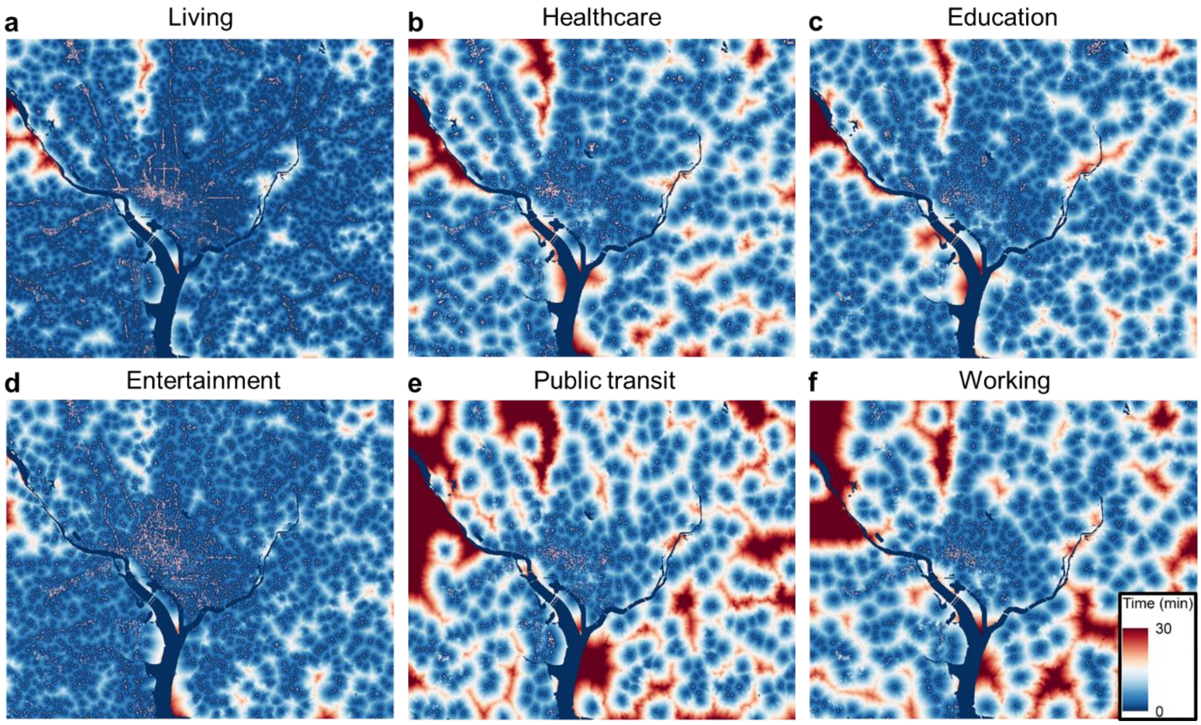

Supplementary Fig. 9. Local example of travel time to living services and amenities with non-motorized transport in Washington, United States. a-f. Travel time maps for living (**a**), healthcare (**b**), education (**c**), entertainment (**d**), public transit (**e**), and working amenities (**f**). Point of interest (POI) datasets are overlaid on the travel time map with purple colours.

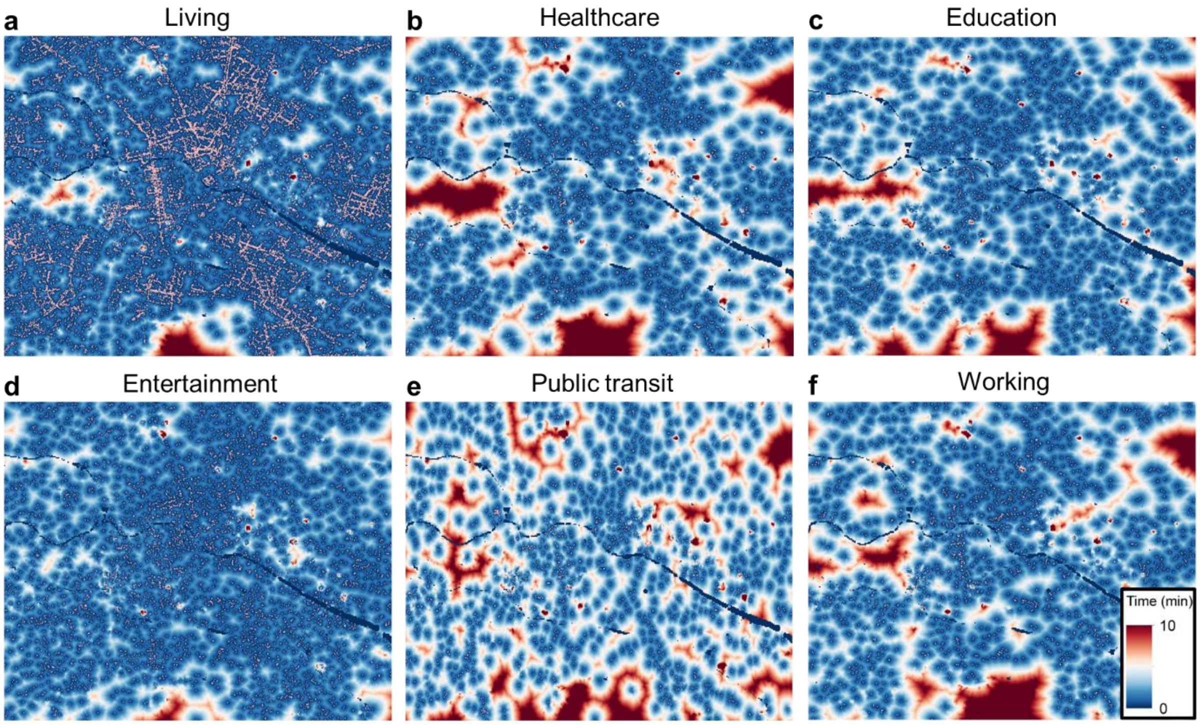

Supplementary Fig. 10. Local example of travel time to living services and amenities with non-motorized transport in Berlin, German. a-f. Travel time maps for living (**a**), healthcare (**b**), education (**c**), entertainment (**d**), public transit (**e**), and working amenities (**f**). Point of interest (POI) datasets are overlaid on the travel time map with purple colours.

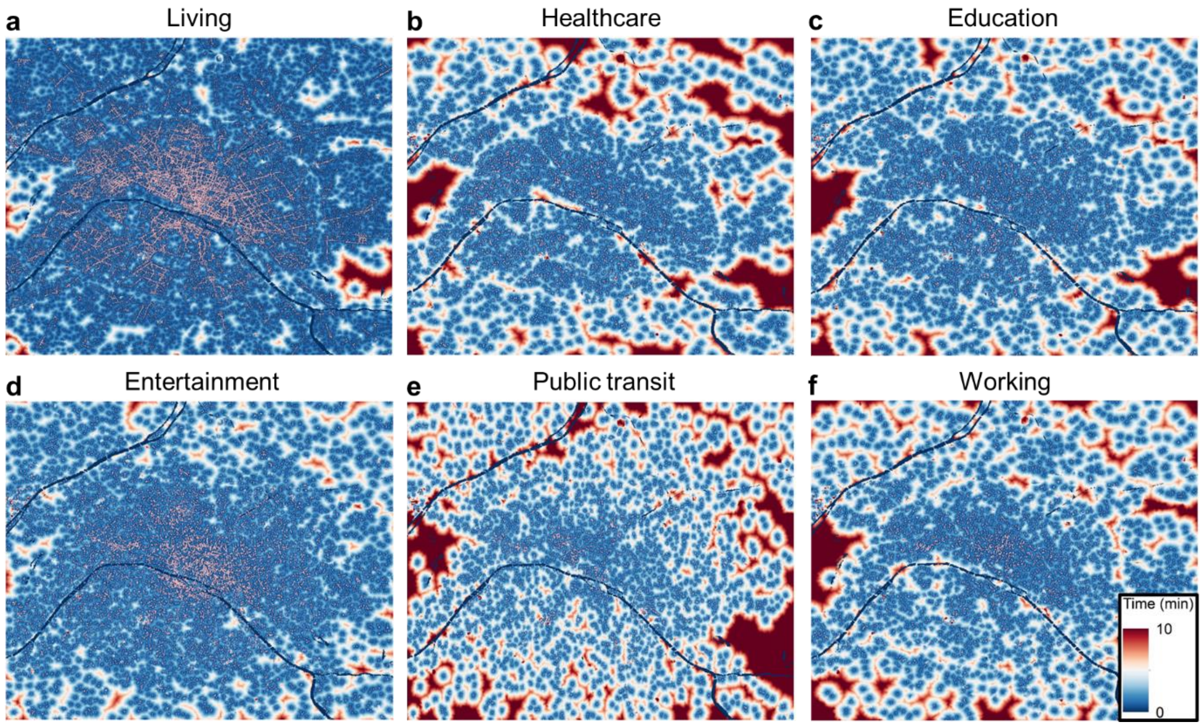

Supplementary Fig. 11. Local example of travel time to living services and amenities with non-motorized transport in Paris, France. a-f. Travel time maps for living (**a**), healthcare (**b**), education (**c**), entertainment (**d**), public transit (**e**), and working amenities (**f**). Point of interest (POI) datasets are overlaid on the travel time map with purple colours.

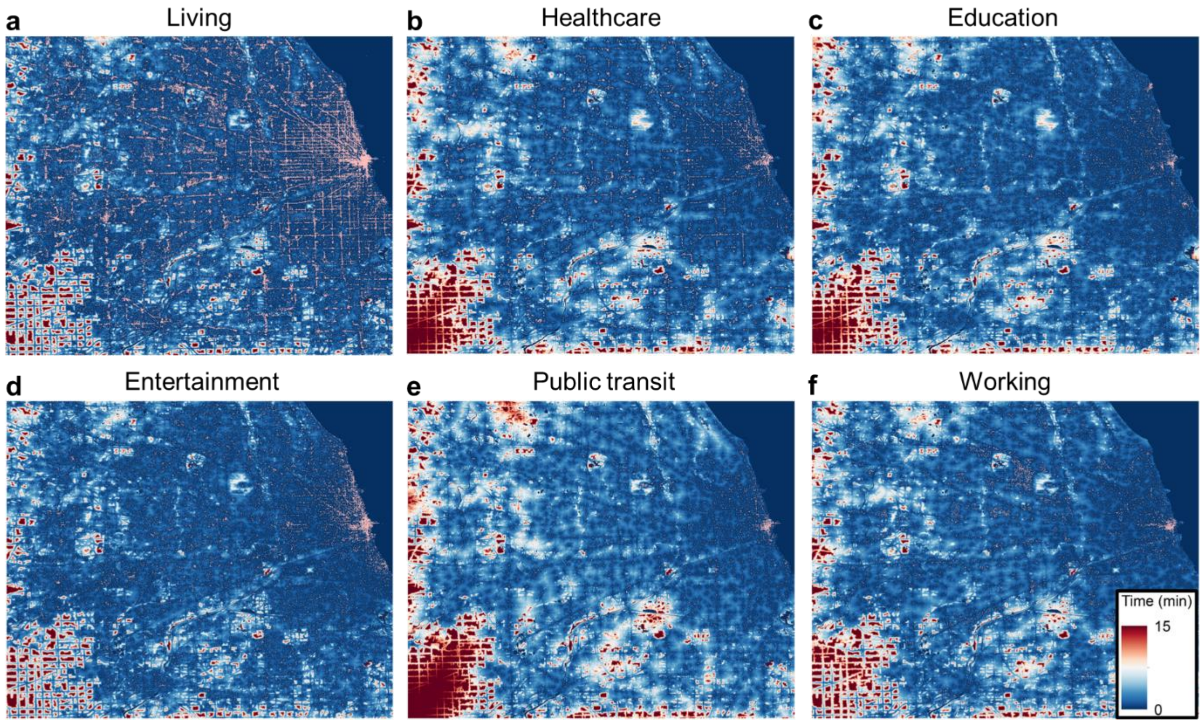

Supplementary Fig. 12. Local example of travel time to living services and amenities with motorized transport in Chicago, United States. a-f. Travel time maps for living (**a**), healthcare (**b**), education (**c**), entertainment (**d**), public transit (**e**), and working amenities (**f**). Point of interest (POI) datasets are overlaid on the travel time map with purple colours.

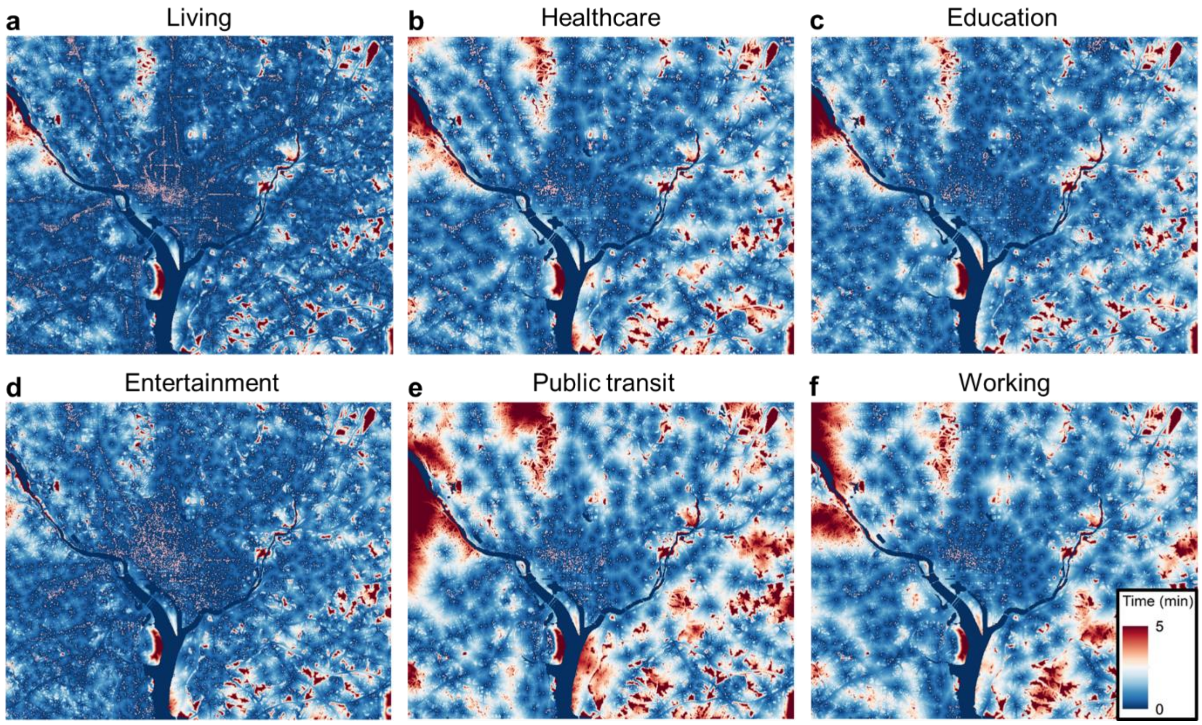

Supplementary Fig. 13. Local example of travel time to living services and amenities with motorized transport in Washington, United States. a-f. Travel time maps for living (**a**), healthcare (**b**), education (**c**), entertainment (**d**), public transit (**e**), and working amenities (**f**). Point of interest (POI) datasets are overlaid on the travel time map with purple colours.

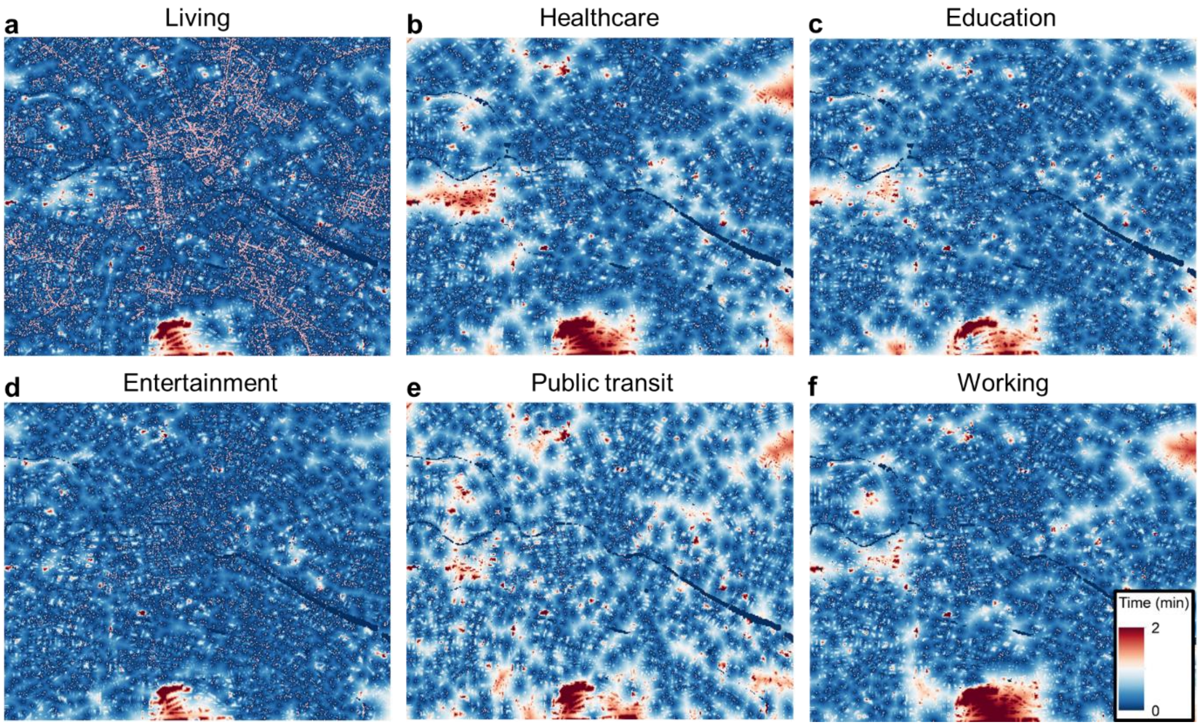

Supplementary Fig. 14. Local example of travel time to living services and amenities with motorized transport in Berlin, German. a-f. Travel time maps for living (**a**), healthcare (**b**), education (**c**), entertainment (**d**), public transit (**e**), and working amenities (**f**). Point of interest (POI) datasets are overlaid on the travel time map with purple colours.

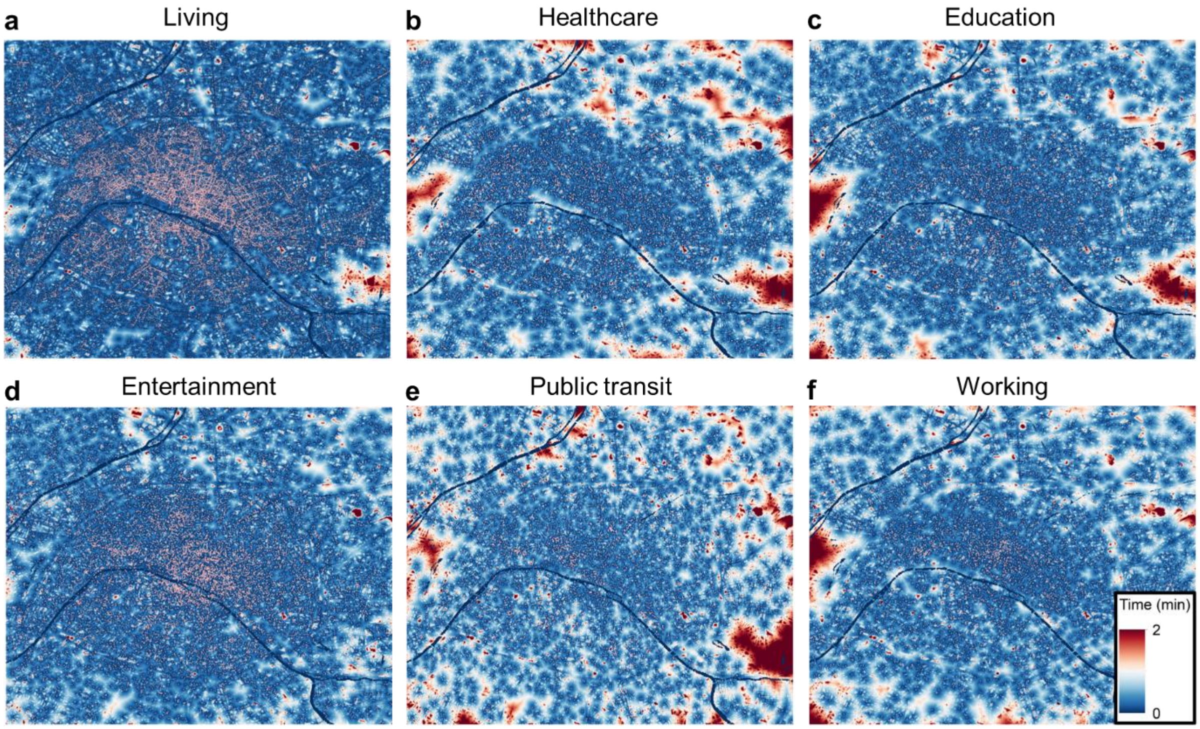

Supplementary Fig. 15. Local example of travel time to living services and amenities with motorized transport in Paris, France. a-f. Travel time maps for living (**a**), healthcare (**b**), education (**c**), entertainment (**d**), public transit (**e**), and working amenities (**f**). Point of interest (POI) datasets are overlaid on the travel time map with purple colours.

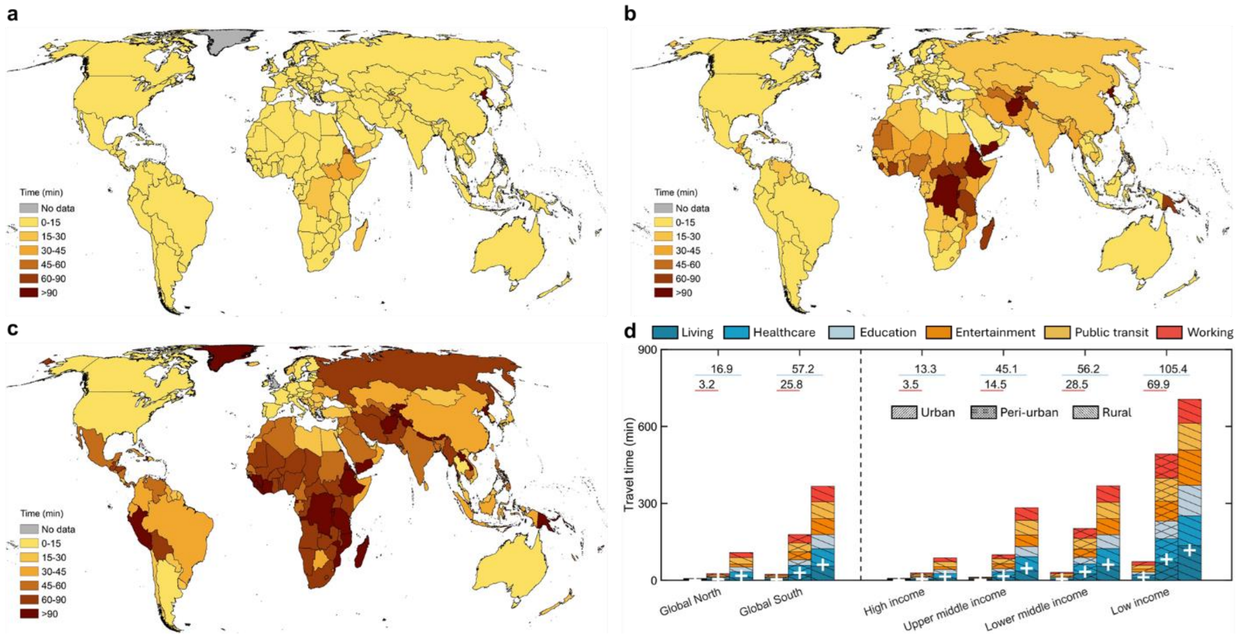

Supplementary Fig. 16. Urban-rural contrasts in resident's travel time to living services and amenities with access to motorized transport. a-c, Maps of mean travel time for urban residents' living services and amenities across urban (**a**), peri-urban (**b**), and rural (**c**) areas, with the associated boundary layers extracted from the degree of urbanization dataset in 2020 from the Global Human Settlement Layer (GHSL) project. **d**, Travel time for six types of living services and amenities by walk across different urbanization (Global North and Global South) and income (high, upper middle, lower middle, and low incomes) contexts. The mean travel time for each social-economic development and income level are shown in each column as "+" symbol in white colour. The mean travel time differences across urban-rural continuum degrees (peri-urban-urban and rural-urban) are also shown. Income group information is from the World Bank database according to 2022 gross national income (GNI) per capita: low income, 1,135 USD or less; lower middle income, 1,136 - 4,465 USD; upper middle income, 4,466 - 13,845 USD; and high income, 13,846 USD or more.

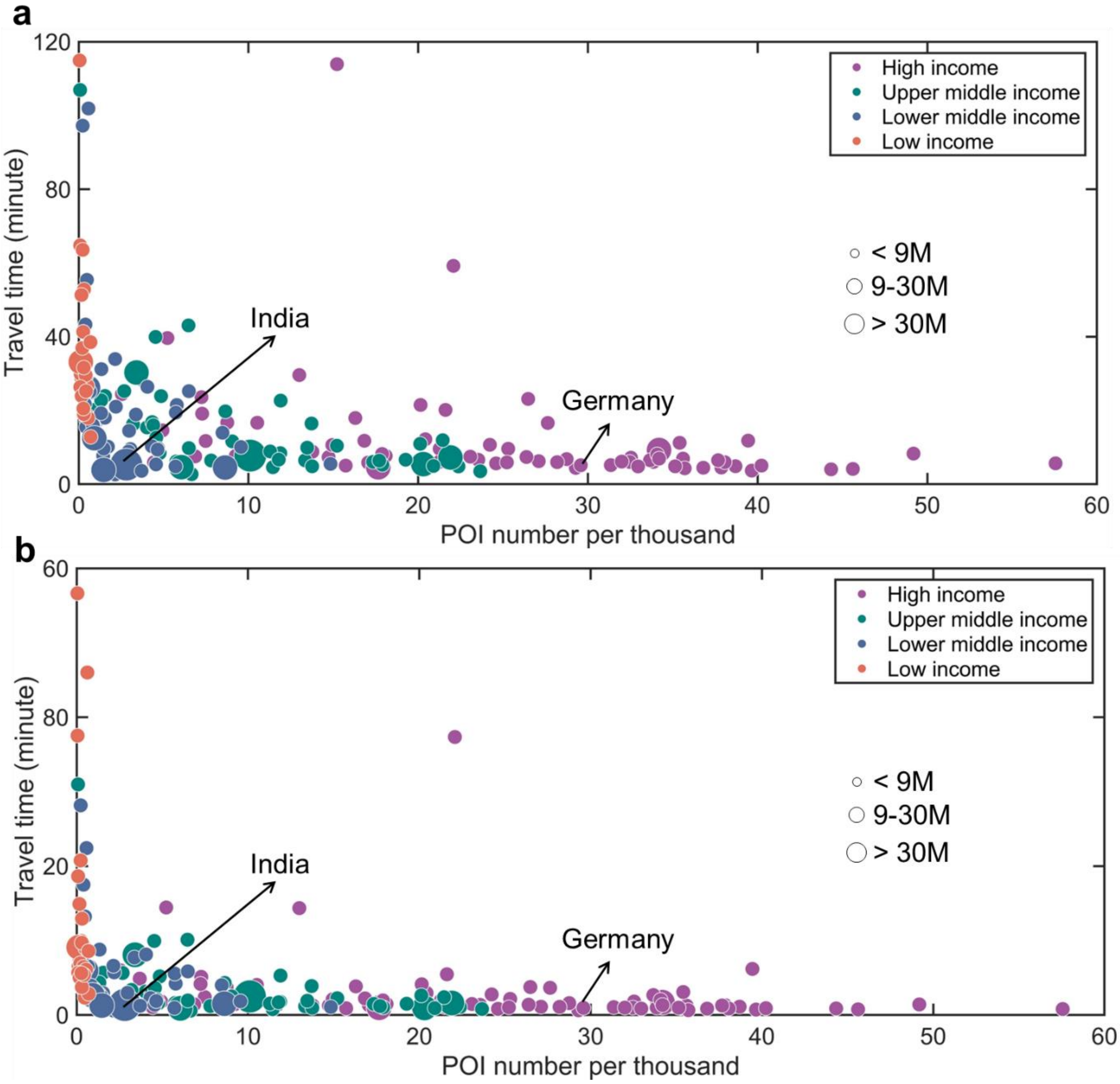

Supplementary Fig.17. Relationship between urban non-motorized (a) and motorized (b) travel time with point of interest (POI) number per thousand across four income levels and three population gradients. Income group information is from the World Bank database according to 2022 gross national income (GNI) per capita: low income, 1,135 USD or less; lower middle income, 1,136 - 4,465 USD; upper middle income, 4,466 - 13,845 USD; and high income, 13,846 USD or more. Population data, extracted from the 100-meter resolution WorldPop dataset, is categorized into three levels: < 9 million, 9 - 30 million, and > 30 million.

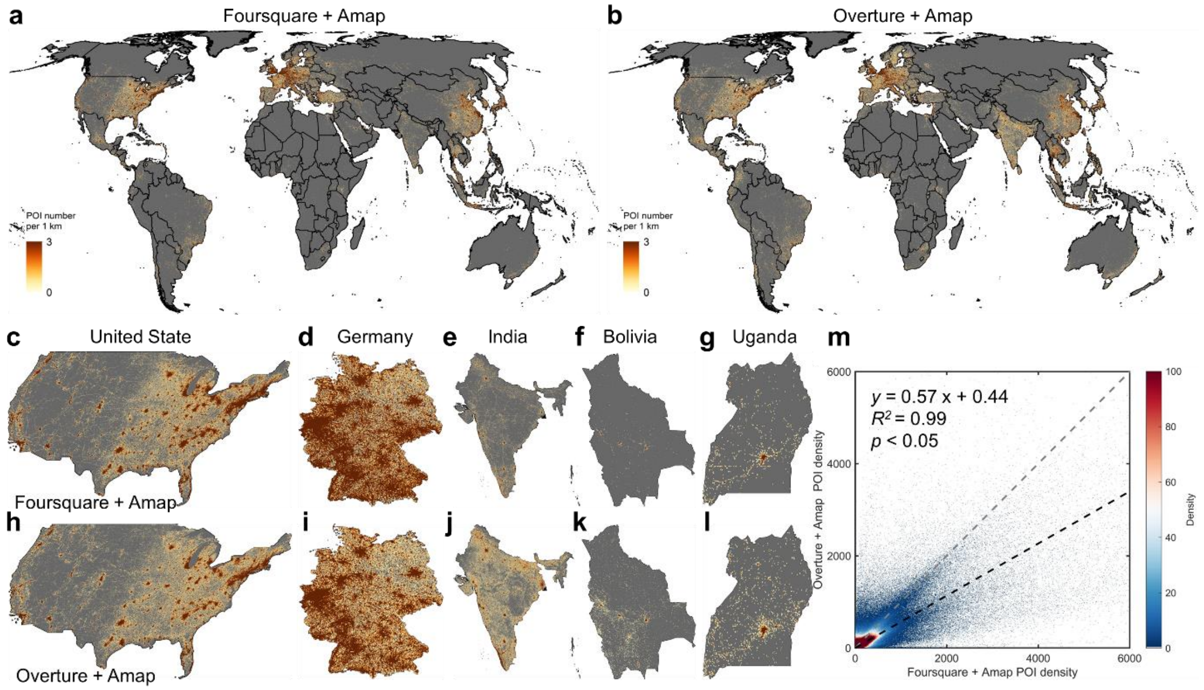

Supplementary Fig. 18. Comparison of point of interest (POI) densities (total POI number per km) between Foursquare + Amap (a, c-f) and Overture + Amap (b, h-l). **a-b.** Global maps of Foursquare + Amap (**a**) and Overture + Amap (**b**) POI densities. **c-l.** Local distributions of POI densities across five example countries: United State (**c** and **h**), Germany (**d** and **i**), India (**e** and **j**), Bolivia (**f** and **k**), and Uganda (**g** and **l**). **m.** Scatter plot of Foursquare + Amap and Overture + Amap POI densities.

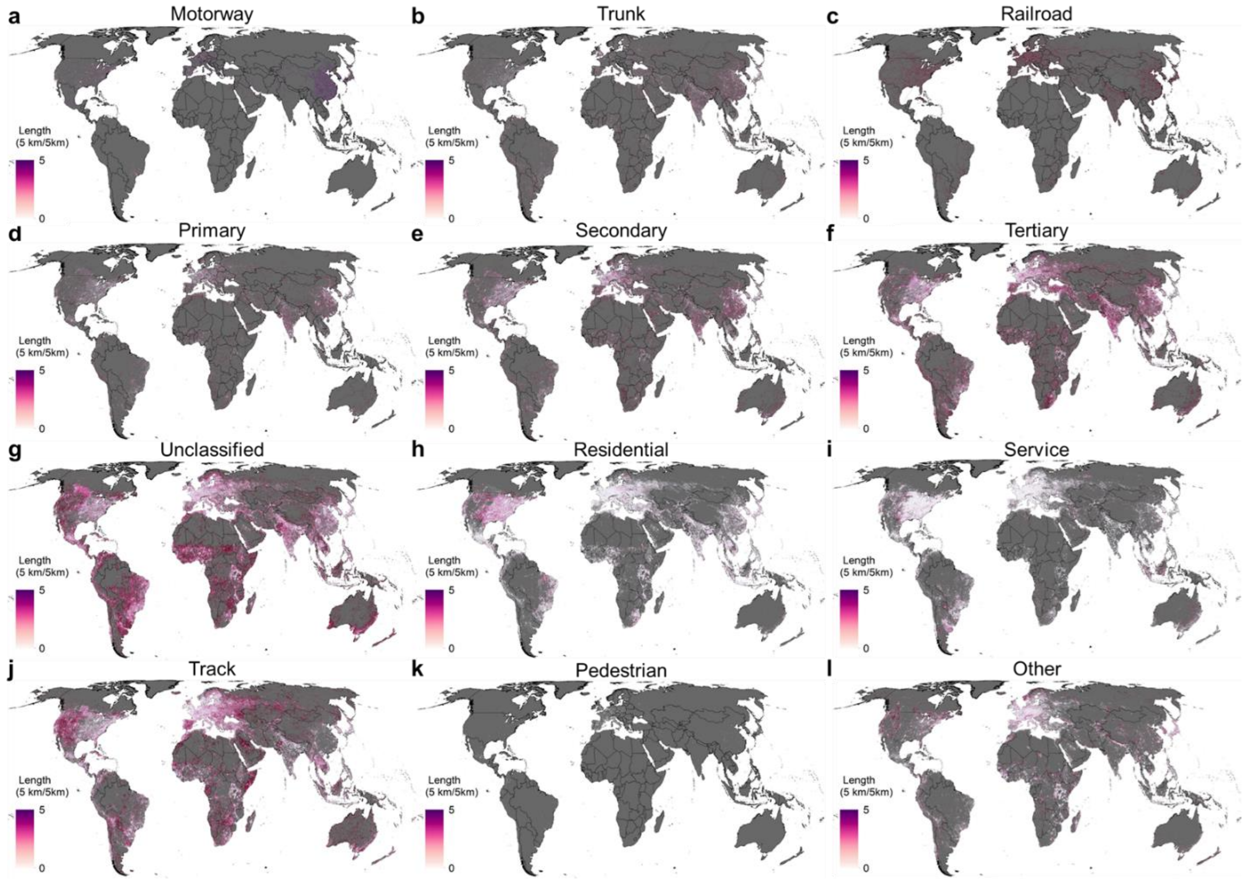

Supplementary Fig. 19. Spatial maps of global road lengths of overture road network by class. **a.** Motorway. **b.** Trunk. **c.** Railroad. **d.** Primary. **e.** Secondary. **f.** Tertiary. **g.** Unclassified. **h.** Residential. **i.** Service. **j.** Track. **k.** Pedestrian. **l.** Other.

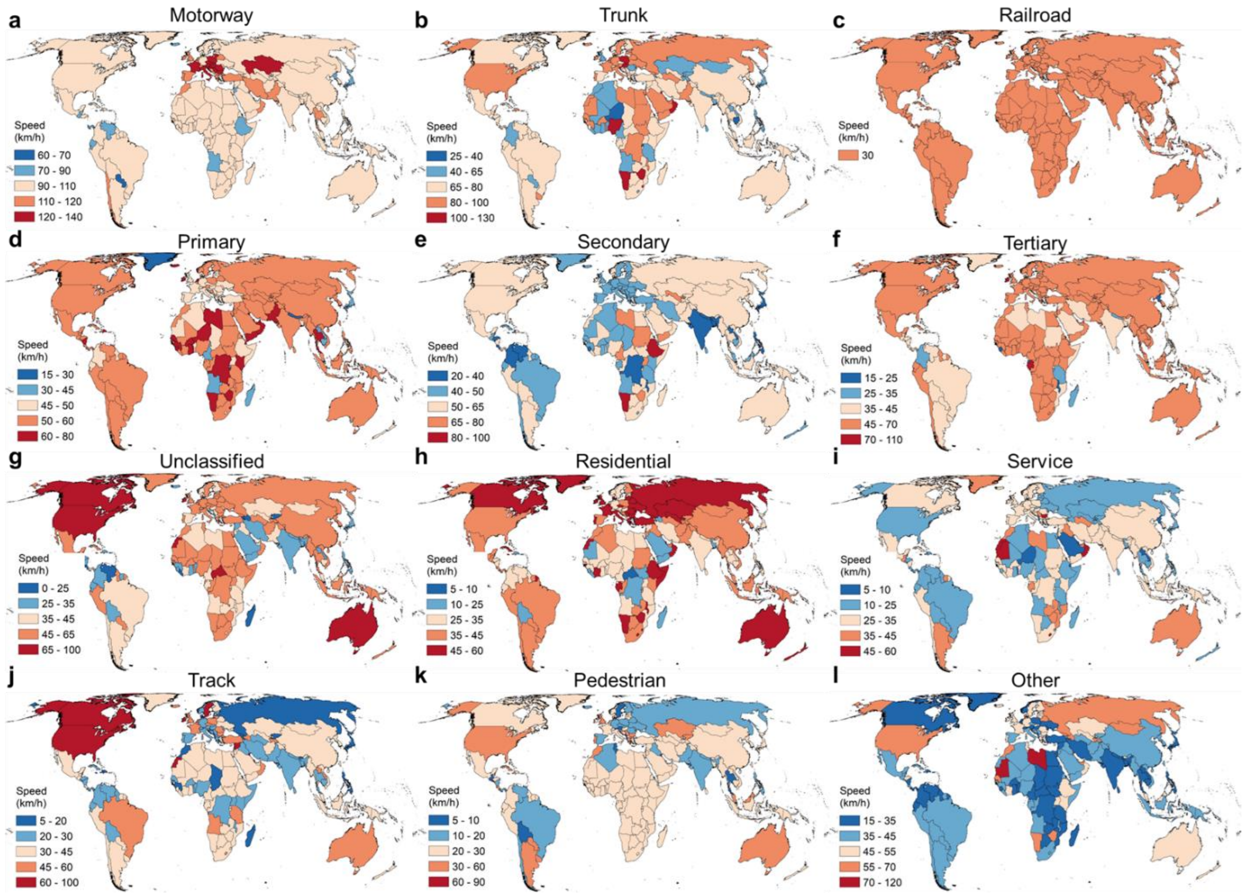

Supplementary Fig 20. Spatial maps of global road speed by class. **a.** Motorway. **b.** Trunk. **c.** Railroad. **d.** Primary. **e.** Secondary. **f.** Tertiary. **g.** Unclassified. **h.** Residential. **i.** Service. **j.** Track. **k.** Pedestrian. **l.** Other. These road speed limitations are adapted from Weiss et al. 2020.

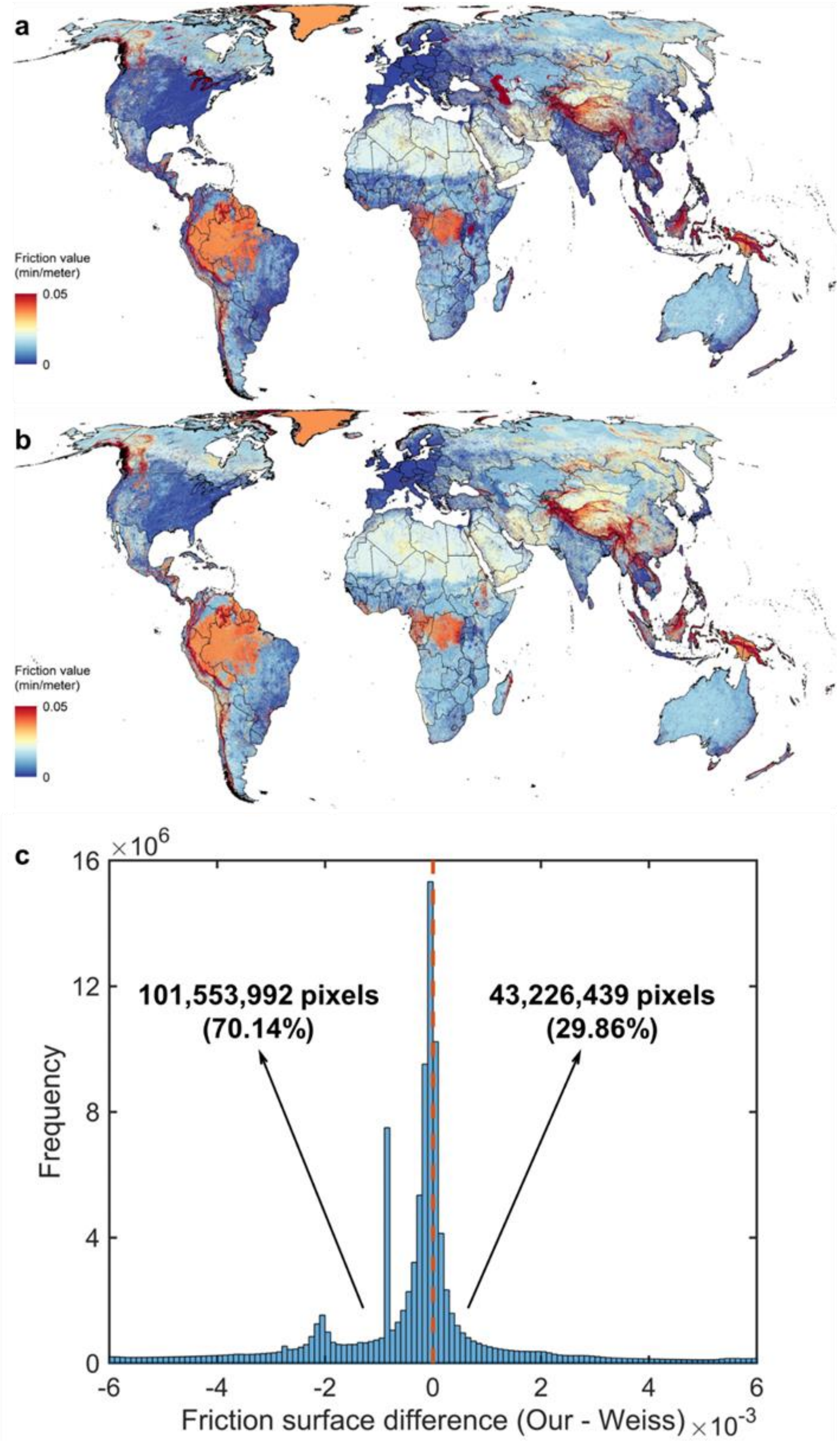

Supplementary Fig. 21. Comparison of 1-km-resolution global friction surface for motorized transport between Overture-based (a) and Weiss's approach (b). a. Friction surface data that is generated from Overture road network together with 500-m-resolution

MODIS MCD12Q1 land cover data and 90-m-resolution GMTED2010 DSM data. **b.** Weiss's friction surface that is generated from OSM and Google road for 2019 together with 500-m-resolution MODIS MCD12Q1 land cover data and 90-m-resolution GMTED2010 DSM data. c. Histogram distribution of the difference between the Overture-based and Weiss's friction surfaces, with statistics inserted in the plots.

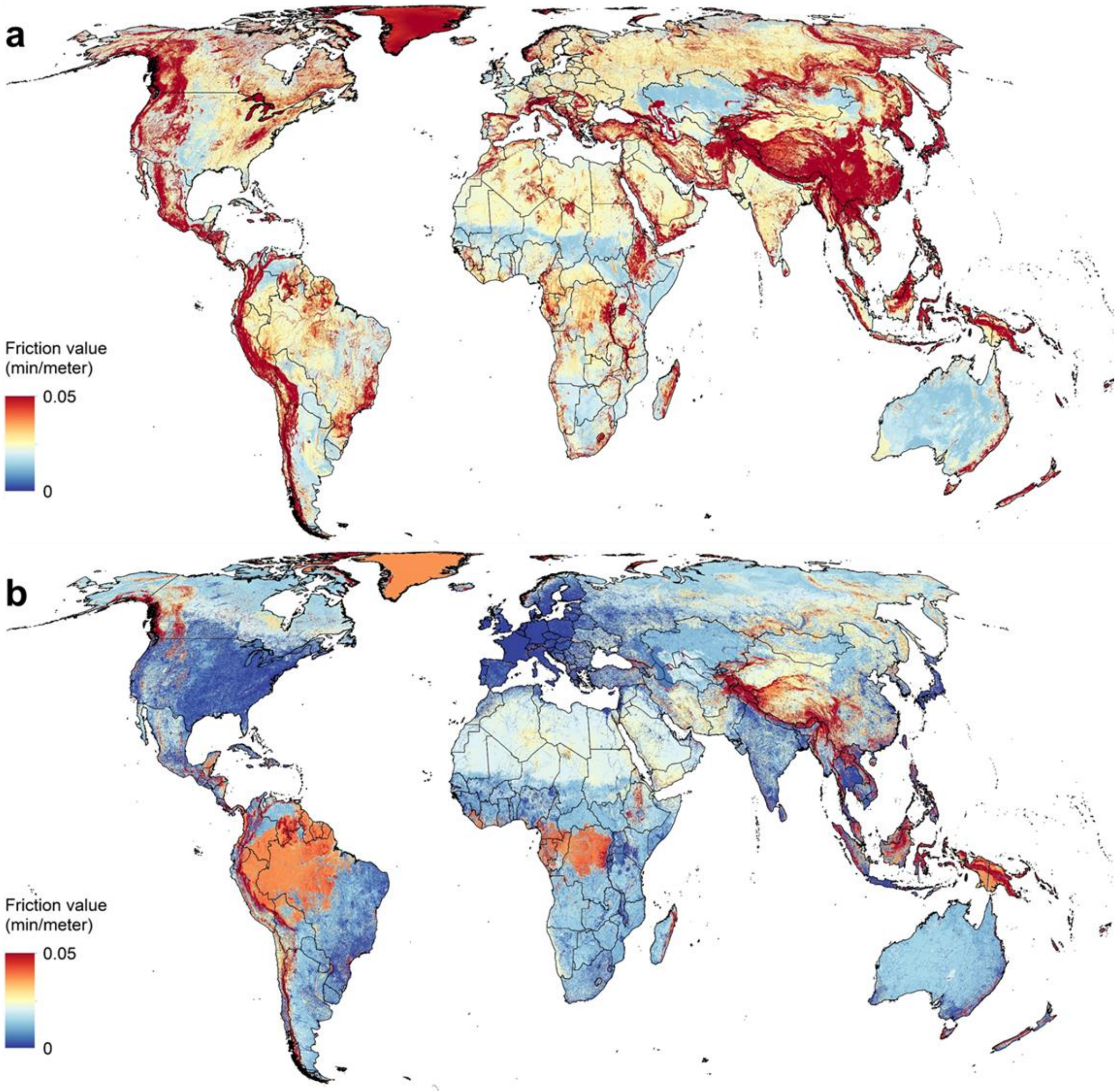

Supplementary Fig. 22. Global maps of friction surface in the non-motorized mode that created by (a) this study, and (b) Weiss's approach. Friction surface in **a** was created from Overture road network, 10-m-resolution WorldCover land cover, and 30-m-resolution ALOS DSM datasets and resampled to 500-m resolution for visualization. Friction surface in **b** was created from OSM and Google Road networks, 500-m-resolution MCD12Q1 land cover, and 30-m-resolution GMTED2010 DSM datasets with a final 1-km data resolution.

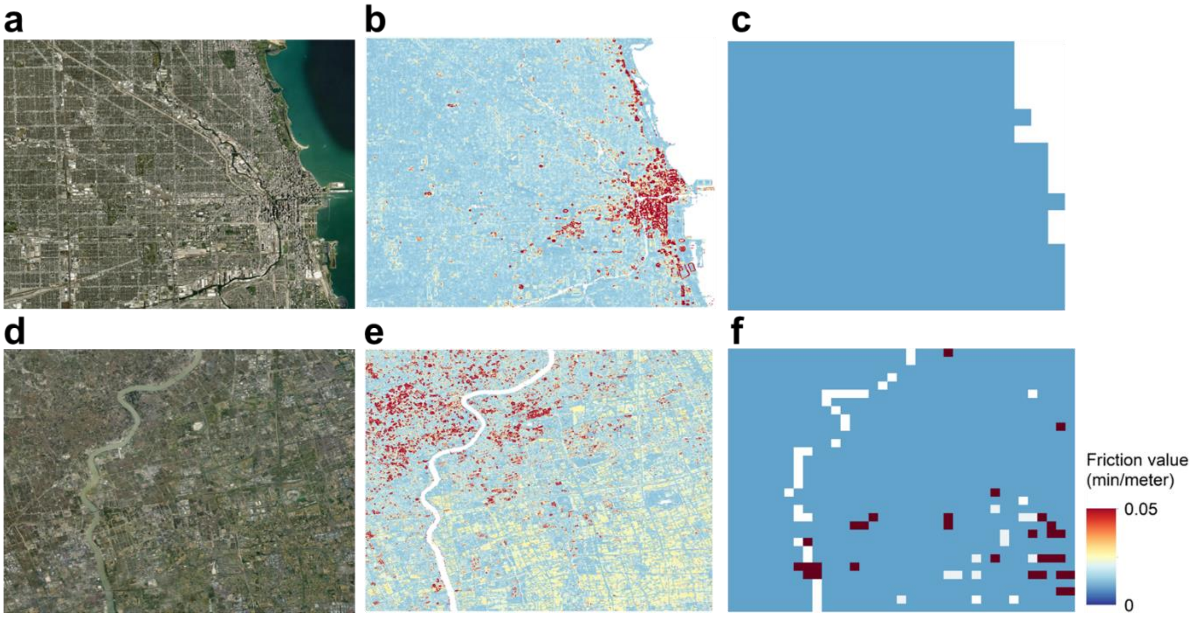

Supplementary Fig. 23. Local examples of friction surface for non-motorized transport generated by (b and e) this study and (c and f) Weiss's approach over (a-c) Chicago, United States and (d-f) Shanghai, China. a, d. Google satellite image. **b**, **e**. Friction surface of this study. **c**, **f**. Friction surface of Weiss's approach.

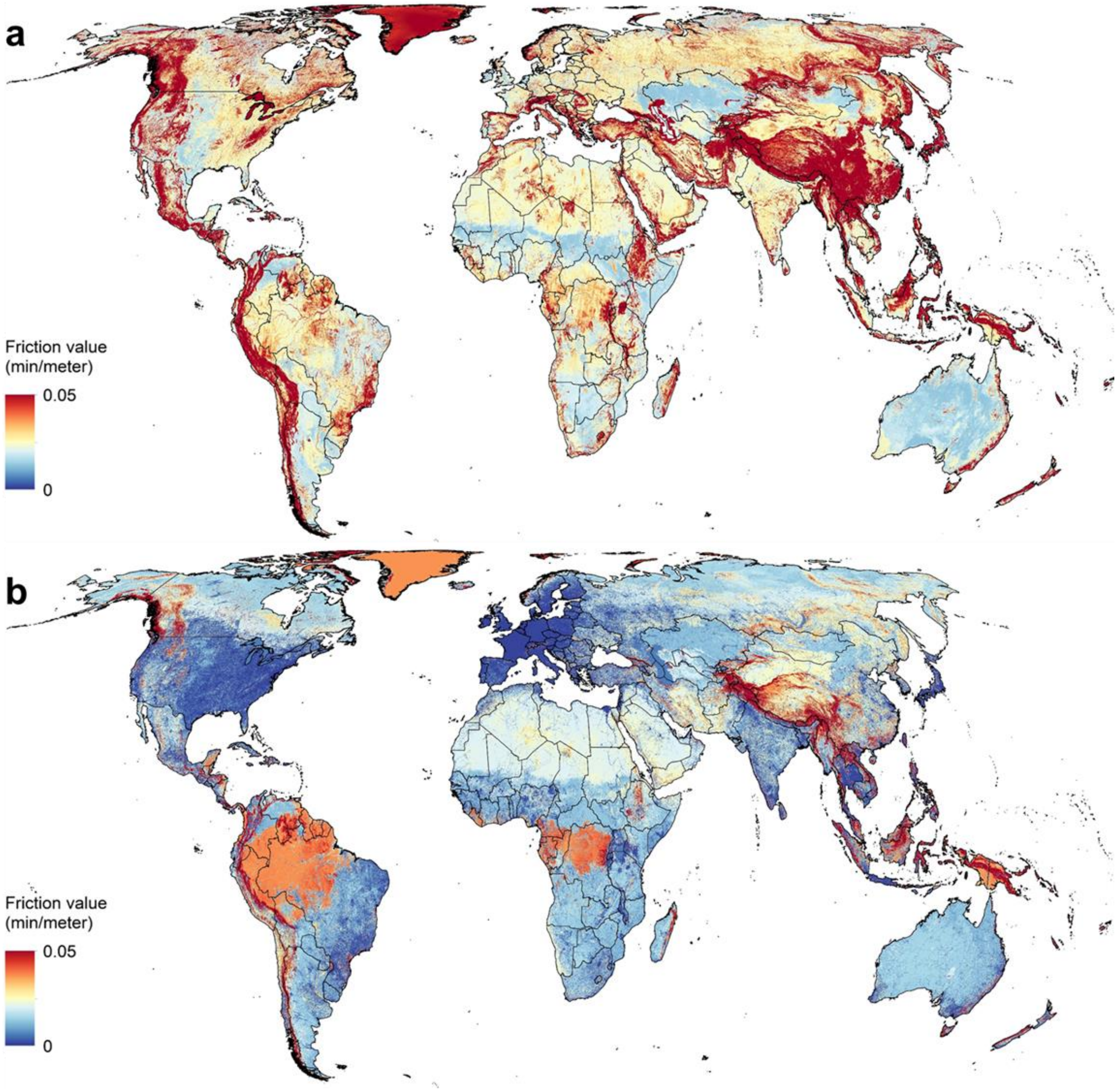

Supplementary Fig. 24. Global maps of friction surface in the motorized mode that created by (a) this study, and (b) Weiss's approach. Friction surface in **a** was created from Overture road network, 10-m-resolution WorldCover land cover, and 30-m-resolution ALOS DSM datasets and resampled to 500-m resolution for visualization. Friction surface in **b** was created from OSM and Google Road networks, 500-m-resolution MCD12Q1 land cover, and 30-m-resolution GMTED2010 DSM datasets with a final 1-km data resolution.

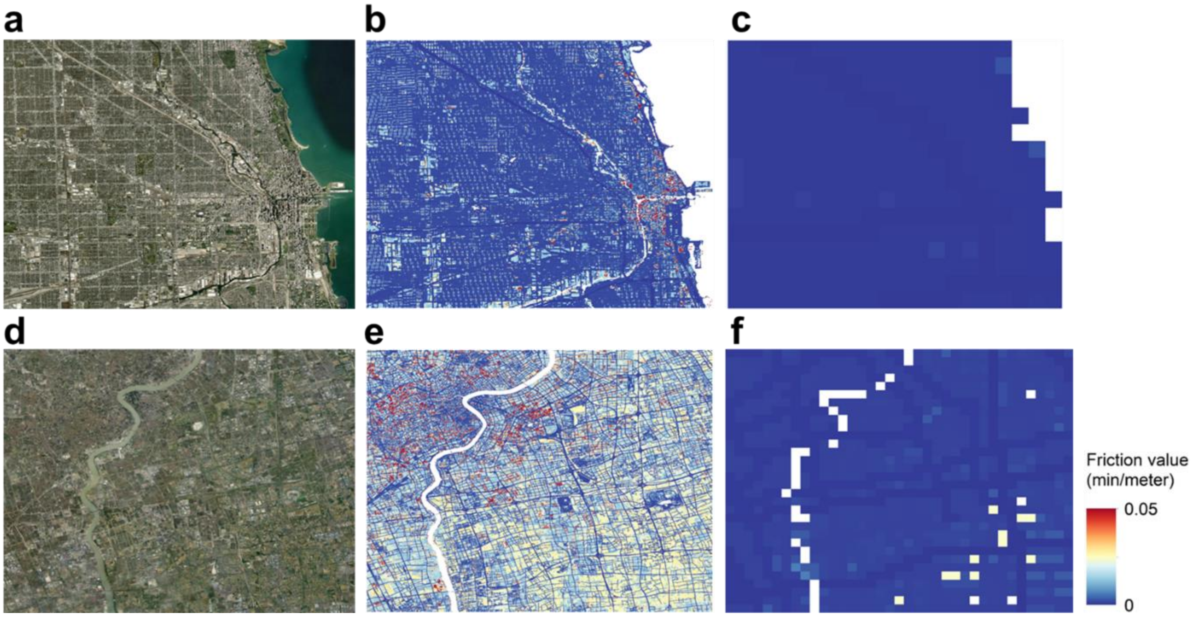

Supplementary Fig. 25. Local examples of friction surface for motorized transport generated by (b and e) this study and (c and f) Weiss's approach over (a-c) Chicago, United States and (d-f) Shanghai, China. a, d. Google satellite image. **b**, **e**. Friction surface of this study. **c**, **f**. Friction surface of Weiss's approach.

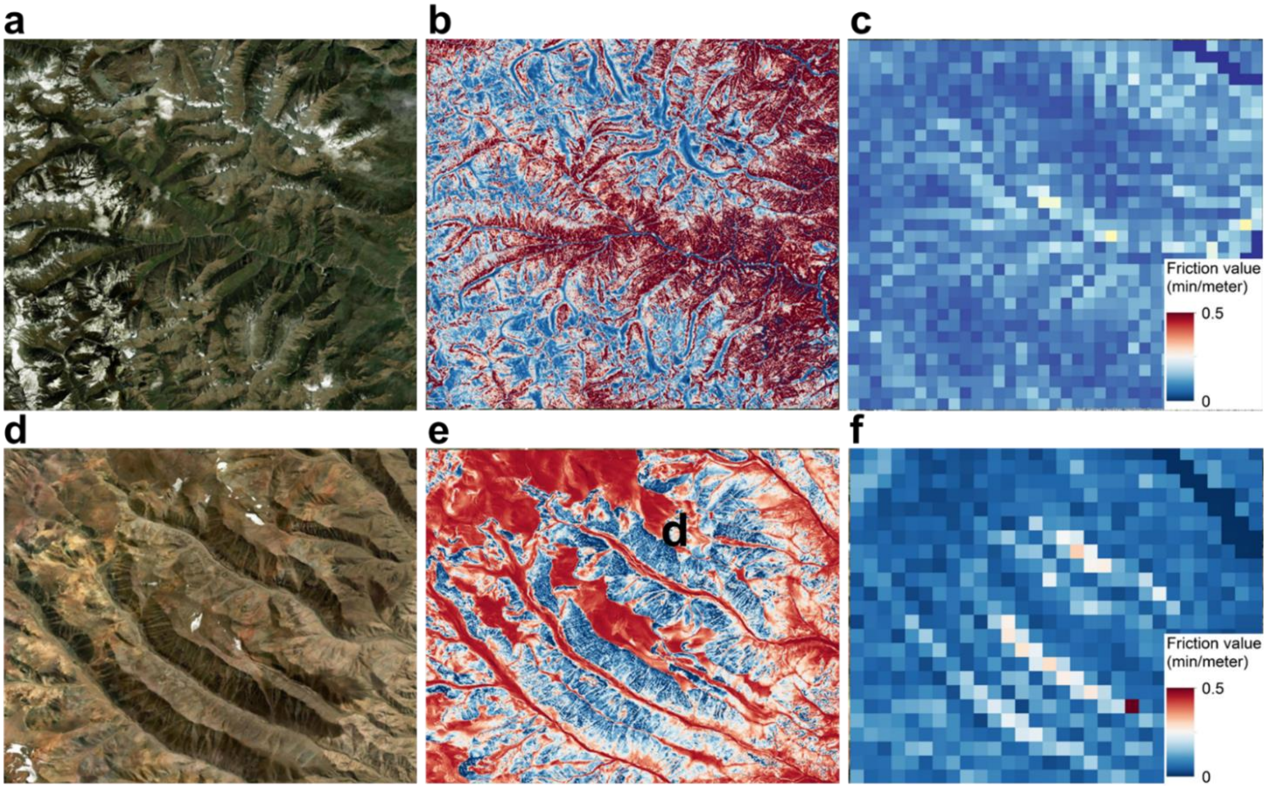

Supplementary Fig. 26. Local examples of friction surface over two mountainous areas. **a-c**. Tibetan plateau Mountain. **d-f.** Andres Mountain. **a**, **d**. Google satellite image. **b**, **e**. Friction surface created in this study. **c**, **f.** Weiss's friction surface map.

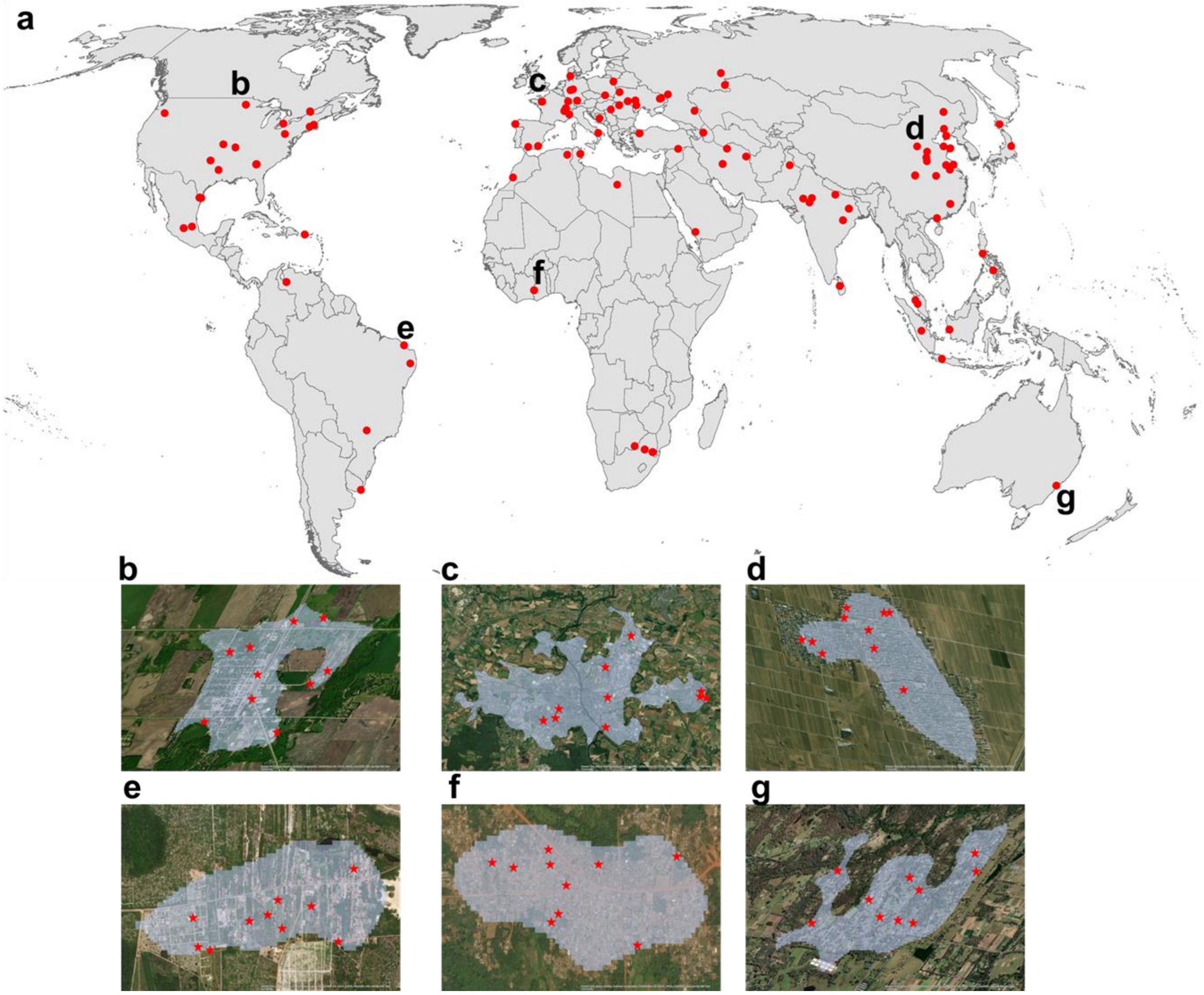

Supplementary Fig. 27. Global sample cities for travel time validation of friction surface-based approach. a. Spatial distribution of 100 sample cities (red points). **b-g.** Local examples of six sample cities with 10 validation points (red pentagrams) for each, which are bounded by google urban boundary (GUB) dataset and overlaid on Google satellite basemaps.

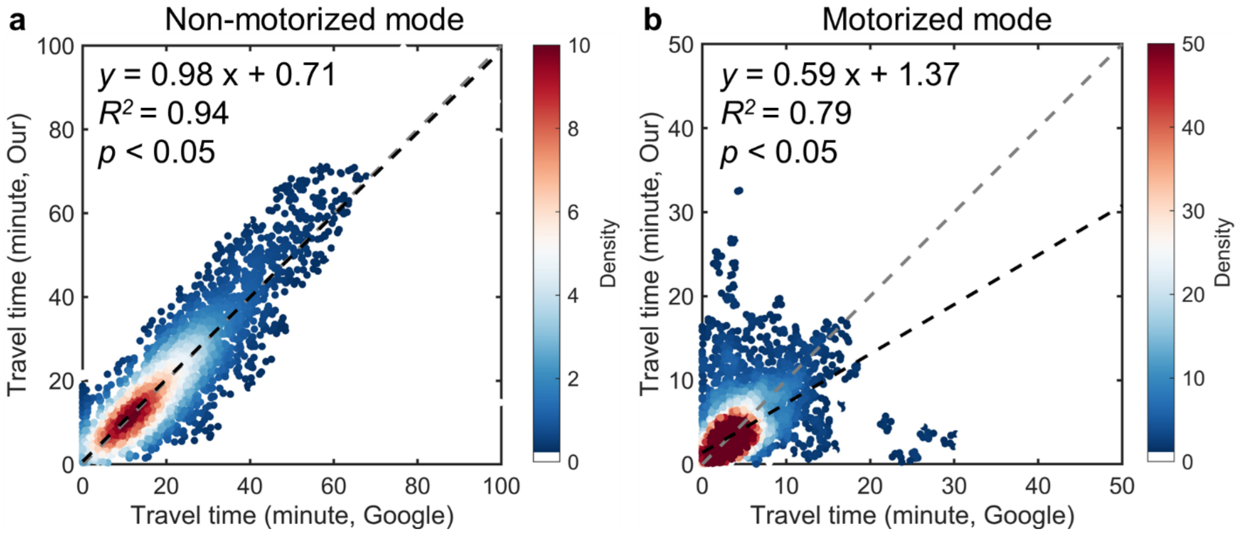

Supplementary Fig. 28. Accuracy validation of travel time for (a) non-motorized and (b) motorized modes. Google travel time is calculated from Google Directions API, and our travel time is calculated from the friction surface with the "least-cost-path" algorithm.

Supplementary Table 1. Classification scheme of six major necessities and services in residents' daily life for the Overture global point of interest (POI) database.

Class	Overture scheme
Living	Eat and drink
Living	Accommodation
Living	Beauty and spa
Living	Financial service
Living	Retail
Living	Home service
Living	Professional services
Healthcare	Health and medical
Education	Education
Entertainment	Arts and entertainment
Entertainment	Attractions and activities
Entertainment	Active life
Public transit	Travel
Working	Business to business

Supplementary Table 2. Classification scheme of six major necessities and services in residents' daily life for the Amap point of interest (POI) database for China. Both English and Chinese classification schemes are listed.

Class	Amap scheme (English)	Amap scheme (Chinese)
Living	Accommodation services	住宿服务
Living	Public utilities	公共设施
Living	Business residence	商务住宅
Living	Domestic services	生活服务
Living	Shopping services	购物服务
Living	Financial insurance services	金融保险服务
Living	Catering services	餐饮服务
Healthcare	Health care services	医疗保健服务
Education	Science, education and cultural services	科教文化服务
Entertainment	Sports and leisure services	体育休闲服务
Entertainment	Famous tourist sites	风景名胜
Public transit	Transportation facilities services	交通设施服务
Public transit	Motorcycle services	摩托车服务
Public transit	Car services	汽车服务
Public transit	Car repair	汽车维修
Public transit	Car sale	汽车销售
Public transit	Access facilities	通行设施
Public transit	Road ancillary facilities	道路附属设施
Working	Company	公司企业

Supplementary Table 3. POI numbers for six major necessities and services.

Class	POI number
Living	41,394,922
Healthcare	4,261,898
Education	4,644,982
Entertainment	6,308,387
Public transit	4,486,765
Working	4,619,809
Total	65,716,763
